# S-acylation of a geminivirus C4 protein is essential for regulating the CLAVATA pathway in symptom determination

**DOI:** 10.1093/jxb/ery228

**Published:** 2018-06-20

**Authors:** Huiyun Li, Runxiu Zeng, Zian Chen, Xiaoshi Liu, Zhendan Cao, Qi Xie, Chengwei Yang, Jianbin Lai

**Affiliations:** 1 Guangdong Provincial Key Laboratory of Biotechnology for Plant Development, School of Life Science, South China Normal University, Guangzhou, China; 2 Institute of Genetics and Developmental Biology, Chinese Academy of Sciences, Beijing, China

**Keywords:** C4, CLAVATA, geminivirus, membrane association, S-acylation, symptom determination

## Abstract

Geminiviruses, such as beet severe curly top virus (BSCTV), are a group of DNA viruses that cause severe plant diseases and agricultural losses. The C4 protein is a major symptom determinant in several geminiviruses; however, its regulatory mechanism and molecular function in plant cells remain unclear. Here, we show that BSCTV C4 is S-acylated *in planta*, and that this post-translational lipid modification is necessary for its membrane localization and functions, especially its regulation of shoot development of host plants. Furthermore, the S-acylated form of C4 interacts with CLAVATA 1 (CLV1), an important receptor kinase in meristem maintenance, and consequentially affects the expression of *WUSCHEL*, a major target of CLV1. The abnormal development of siliques in *Arabidopsis thaliana* infected with BSCTV is also dependent on the S-acylation of C4, implying a potential role of CLAVATA signaling in this process. Collectively, our results show that S-acylation is essential for BSCTV C4 function, including the regulation of the CLAVATA pathway, during geminivirus infection.

## Teaser

S-acylation of the beet severe curly top virus C4 protein is essential for its membrane association, interaction with CLV1, and symptom determination during geminivirus infection.

## Introduction

Geminiviruses are a family of plant viruses with circular single-stranded DNA genomes that cause plant diseases worldwide. Geminivirus-encoded proteins regulate host cellular processes, including DNA replication, gene silencing, and hormone signaling, to complete their infection cycle (Ramesh *et al.*, 2017). The cell cycle is an important process targeted by geminiviruses (Gutierrez, 1999); for instance, the geminivirus Rep protein binds to RETINOBLASTOMA-RELATED, a critical plant cell cycle regulator, and thereby releases the transcription factor E2F for virus replication (Arguello-Astorga *et al.*, 2004). Although the Rep-related proteins are essential for virus replication, the abnormal cell division of host plants infected with many geminiviruses is induced by symptom determinants (Hanley-Bowdoin *et al.*, 2013). Understanding the regulatory mechanisms of symptom determinants is therefore important for developing strategies to protect plants from geminiviruses.

The conserved C4 (or L4) proteins in monopartite geminiviruses and AC4 (or AL4) proteins in bipartite geminiviruses have important functions (Deom and Mills-Lujan, 2015). The disruption of C4 reduces symptom development in many geminivirus infections (Stanley and Latham, 1992; Teng *et al.*, 2010), suggesting it is a major determinant of pathogenesis. The expression of *C4* genes from several geminiviruses, including beet curly top virus (BCTV) and beet severe curly top virus (BSCTV), results in ectopic cell division in *Arabidopsis thaliana* and *Nicotiana benthamiana* (Latham, 1997; Piroux *et al.*, 2007; Mills-Lujan and Deom, 2010), supporting the idea that the leaf-curling and vein-swelling symptoms are caused by an abnormal cell cycle. Previous studies have indicated that BSCTV C4 induces the expression of *RKP* for cell cycle regulation (Lai *et al.*, 2009) and *ATHB7/12* for infected cell development (Park *et al.*, 2011), consistent with its effects on cell division. The C4 proteins have been shown to interact with SHAGGY-like protein kinases (Piroux *et al.*, 2007) and affect the brassinosteroid (BR) pathways (Mills-Lujan and Deom, 2010; Bi *et al.*, 2017). In addition, the C4 proteins of some geminiviruses are involved in virus movement and gene silencing (Ramesh *et al.*, 2017). Despite these recent discoveries, the precise molecular mechanisms by which C4 contributes to symptom determination, as well as how C4 is regulated in the host cells, remain unclear.

Phosphorylation and myristoylation have been reported to regulate the function of C4 proteins in pathogenesis (Fondong *et al.*, 2007; Piroux *et al.*, 2007; Mills-Lujan *et al.*, 2015); however, it is unclear whether other regulatory mechanisms contribute to the function of C4 during geminivirus infection. Because BSCTV is able to efficiently infect model plants such as Arabidopsis and *N. benthamiana*, causing severe symptoms (Park *et al.*, 2002), it provides a powerful system to study the interactions between geminiviruses and hosts. Here, we determined that the C4 protein from BSCTV is S-acylated in plant cells. S-acylation is a type of lipid modification that transfers a long-chain fatty acid such as palmitate to the cysteine residues of proteins (Linder and Deschenes, 2007). Unlike myristoylation, which is irreversible, S-acylation is a reversible modification that regulates the subcellular localization, stability, and activity of the substrates, and is important for many biological processes in a variety of plant species (Li and Qi, 2017); for example, S-acylation regulates the membrane association of the RHO family proteins (Lavy *et al.*, 2002) and calcineurin B-like proteins (CBLs) (Batistič *et al.*, 2012) for signaling transduction. Recently, S-acylation was reported to be involved in the regulation of leaf senescence, stress tolerance, and root hair development (Hemsley *et al.*, 2005; Zhou *et al.*, 2013; Lai *et al.*, 2015), but its functions in plant–virus interactions remain unknown.

## Materials and methods

### Plant material and growth conditions

Seeds of *Arabidopsis thaliana* Columbia-0 (wild-type, WT) were surface-sterilized for 2 min in 75% ethanol followed by 5 min in 1% NaClO solution, rinsed five times with sterile water and plated on Murashige and Skoog (MS) medium with 1.5% sucrose and 0.8% agar. They were then stratified at 4 °C in the dark for 2 d and subsequently grown under long-day conditions (16/8 h of light/dark) at 22 °C.

### Protoplast transformation and confocal microscopy

For transient expression in protoplasts, the coding sequence (CDS) of *C4* was cloned into a *pBluscript*-based vector under the *35S* promoter, fused with yellow fluorescent protein (YFP), 3× FLAG, and 6× histidines (Liu *et al.*, 2016). The C8S mutation version of this plasmid was generated by PCR using a 5′-primer harboring the mutation site. The plasmids expressing different versions of C4 proteins were transformed into Arabidopsis protoplasts as previously described (Yoo *et al.*, 2007). The transformed protoplasts were incubated for 18 h at 22 °C, and the subcellular localization of YFP-fused proteins was observed under a Zeiss LSM 710 laser-scanning microscope (514 nm for excitation and 530–600 nm for emission).

### Acyl-RAC assays

Acyl-resin-assisted capture (Acyl-RAC) assays were performed following the protocol previously described by Lai *et al.* (2015). In particular, the plasmids expressing C4^WT^-YFP-FLAG_3_His_6_ or C4^C8S^-YFP-FLAG_3_His_6_ were transformed into protoplasts as described by Yoo *et al.* (2007). After incubation for 48 h (or other specified times), the cells were collected and lysed in lysis buffer (25 mM HEPES, 25 mM NaCl, 1 mM EDTA, pH 7.5) containing a protease inhibitor cocktail. Equal amounts of proteins were diluted in blocking buffer (100 mM HEPES, 1.0 mM EDTA, 2.5% SDS, 0.5% MMTS, pH 7.5) and incubated at 40 °C for 10 min with frequent vortexing. Three volumes of cold acetone were added, and the sample was allowed to precipitate at −20 °C for 20 min. The precipitated proteins were collected by centrifugation at 5000 *g* for 10 min, and the pellet was washed with 70% acetone, re-suspended in 300 µl of binding buffer (100 mM HEPES, 1.0 mM EDTA, 1% SDS, pH 7.5) and mixed with 40 µl of pre-washed Thiopropyl Sepharose 6B (Sigma). Then, 40 µl of either 2 M NH_2_OH (pH 7.5) or 2 M NaCl (control) was added into this mixture. The mixtures were rotated at room temperature for 2 h and 20 µl of each supernatant was saved as the total input. Resins were washed five times with binding buffer. Elution was performed using 60 µl of binding buffer containing 50 mM DTT at room temperature for 20 min. Supernatants were removed and mixed with protein sample buffer, incubated at 95 °C for 5 min, and then used for SDS-PAGE and immunological detection.

### Cell fraction assays

The wild-type or mutant versions of *C4-YFP-FLAG*_*3*_*His*_*6*_ were transiently expressed in protoplasts. At 48 h after transformation, the protoplasts were collected for cell fraction as previously described (Mei *et al.*, 2018) with minor modification. The cells were re-suspended in homogenization buffer (50 mM Tris pH 7.4, 150 mM NaCl, 1 mM EDTA, 13% sucrose with protease inhibitor). The samples were centrifugated at 3000 *g* for 20 min at 4 °C to remove nuclei and large cellular debris, and the supernatant was ultra-centrifuged at 50000 *g* for 1 h at 4 °C to generate soluble and pellet fractions. The pellet fraction was re-suspended in homogenization buffer. All fractions were then used for immunoblot analysis.

### Generation of transgenic plants

For inducible expression of BSCTV C4 in Arabidopsis, the *pER8-C4* plasmid described previously was used (Lai *et al.*, 2009). To generate the C8S mutation versions of this plasmid, a 5′-primer harboring the mutation site was used in PCR amplification. The constructs were transformed into *Agrobacterium EHA105*, which was then used to transform wild-type Arabidopsis (Columbia) by the floral-dip method (Clough and Bent, 1998). The seeds were germinated on media including DMSO or 2 µM estradiol (Sigma), or germinated on regular MS medium for 7d and transferred onto media with or without 2 µM estradiol for an additional 3 weeks.

### Transient expression of proteins in *Nicotiana benthamiana* leaves

For transient expression in *N. benthamiana*, the wild-type or mutant version of *C4* was cloned into the *pCanG-MYC* vector and fused with a MYC tag at its C terminus under the *35S* promoter. Leaves of *N. benthamiana* were infiltrated via *A. tumefaciens* as described by Liu *et al.* (2010). At 4 d after infiltration, the leaves were imaged and total protein was extracted for immunological blotting.

### Virus inoculation

For construction of the BSCTV plasmids, the wild-type virus with 1.8 copies of the BSCTV genome in the *pCAMBIA1300* vector was used, as previously described (Lai *et al.*, 2009). To generate the virus carrying the C8S mutant on C4 (which did not alter the amino acid sequence of the overlapping Rep protein), the 0.8 copy and 1 copy of the virus genome were separately cloned into *pET28a* as an intermediate vector, and the mutation was introduced by site-directed mutagenesis. These mutant fragments were then used to replace the wild-type version of the BSCTV genome sequentially in *pCAMBIA1300*. The insertion direction and sequence information were confirmed by digestion and DNA sequencing. The constructs were transformed into *A. tumefaciens EHA105*. The agrobacteria were resuspended and adjusted to OD_600_=1, and then serial dilutions were used for inoculation on the leaves of *N. benthamiana* or Arabidopsis plants (Teng *et al.*, 2010). Symptoms occurred around 9 d after inoculation.

### DNA gel blot analysis

For the DNA gel blot analysis, total DNA was extracted using the CTAB method from protoplasts or *N. benthamiana* tissues after BSCTV infection. Total genomic DNA was separated by electrophoresis in 0.8% agarose gels and transferred to a Hybond N+ membrane. A whole-genome fragment of BSCTV, digested from *pCambia-1300-BSCTV* with *Eco*RI, was used as a probe (Teng *et al.*, 2010). Probe labeling and signal detection were performed with a DIG-High Prime DNA Labeling and Detection Starter Kit II (Roche).

### Yeast two-hybrid assays

The CDS of *C4* was cloned into *pGBKT7* and the intracellular catalytic domain of CLV1 (704AA-967AA) was cloned into *pGADT7*. Yeast two-hybrid (Y2H) assays were performed using the Matchmaker GAL4-based Two-Hybrid System 3 (Clontech) according to the manufacturer’s instructions. Interactions were tested by stringent selection (SD/–Leu/–Trp/–His) supplied with 1 mM 3-amino-1,2,4-triazole (Sigma). Yeast cells with *pGBKT7-p53* and *pGADT7-T* (Clontech) were used as positive controls; the yeast cells with *pGBKT7-Lam* and *pGADT7-T* (Clontech) were used as negative controls.

### BiFC assays

The wild-type or mutant version of *C4* was cloned into the *pSPYCE-35* vector, while *CLV1* was cloned into the *pSPYNE-35S* vector (Walter *et al.*, 2004). The plasmids were co-transformed into Arabidopsis protoplasts. Combinations with empty vectors were used as negative controls. Bimolecular fluorescence complementation (BiFC) assays were performed as previously described (Walter *et al.*, 2004). YFP signals were detected under a Zeiss LSM 710 laser-scanning microscope.

### Co-Immunoprecipitation assays

For expression of CLV1-MYC in protoplasts, the full length CDS of *CLV1* was cloned into *pCanG-MYC* by homologous recombination. Using *pCanG-MYC* alone as a control, *pCanG-CLV1-MYC* was co-transformed with the wild-type or mutant version of *35S::C4-YFP-FLAG*_*3*_*His*_*6*_ into protoplasts (Yoo *et al.*, 2007). At 48 h after transformation, the protoplasts were collected for co-immunoprecipitation (Co-IP) assays. Proteins were extracted in extraction buffer (10 mM Tris-HCl, pH 7.4, 100 mM NaCl, 10% glycerol, and 1% DDM) containing a protease inhibitor cocktail (Roche). After centrifugation at 13000 *g* for 10 min, the supernatant was incubated with anti-MYC (Cell Signaling Technology) antibody at 4 °C overnight, and rotated with the protein A resin for another 3 h. Then the resins were centrifuged and washed three times with washing buffer (10 mM Tris-HCl, pH 7.4, 100 mM NaCl, 10% glycerol, and 0.1% DDM). Proteins were eluted with SDS sample buffer and analysed by immunoblotting using anti-GFP (green fluorescent protein; Abcam) or anti-MYC (Cell Signaling Technology) antibodies. For Co-IP between CLV1-GFP and C4-MYC, the reagents and protocol were similar except that the GFP-Trap resin (Chromotek) was used instead.

### GUS staining

For detection of the expression of *WUS* and *CLV3*, the *WUSpro::GUS-GFP* or *CLV3pro::GUS-–GFP* construct (previously described by Cui *et al.*, 2015) was introduced into *pER8-C4*^*WT*^ or *pER8-C4*^*C8S*^ by genetic crossing. After growth on medium containing 2 µM estradiol for a specified time, the seedlings were sampled for GUS (β-glucuronidase) staining. The GUS activity analysis was performed according to the method described by De Schutter *et al.*, 2007.

## Results

### S-acylation of C4 in plant cells is essential for its membrane localization

**Fig. 1.**
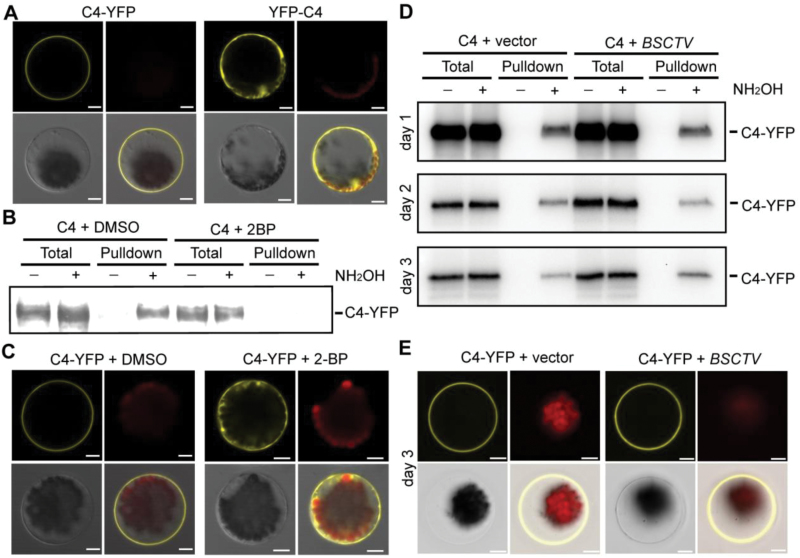
S-acylation of BSCTV C4 in plant cells is essential for its membrane localization. (A) *C4-YFP-FLAG*_*3*_*His*_*6*_ or *YFP-C4* were expressed in protoplasts and fluorescence was detected by confocal microscopy 18 h after transformation. The YFP signal (yellow), chloroplast autofluorescence (red), bright field (gray), and merged images are shown. Scale bars are 10 µm. (B) Detection of C4 S-acylation in plant cells. C4-YFP-FLAG_3_His_6_ was expressed in protoplasts (treated overnight with DMSO or 20 µM S-acylation inhibitor 2-BP) for acyl-resin-assisted capture assays. The signals from the total lysates are shown as the input control, and pulldown indicates the S-acylation proteins captured on the Thiopropyl Sepharose. S-acylation pulldown is dependent on NH_2_OH. The signals were detected using immunological blotting with an anti-FLAG antibody. (C) *C4-YFP-FLAG*_*3*_*His*_*6*_ was expressed in protoplasts with or without a treatment with 20 µM 2-BP. The fluorescence signals (yellow) were detected using confocal microscopy. Scale bars are 10 µm. (D) S-acylation of C4-YFP-FLAG_3_His_6_ was detected in protoplasts without (vector) or with BSCTV infection. The levels of S-acylation were measured 1, 2, and 3 d after infection. (E) Representative images of localization of C4-YFP-FLAG_3_His_6_ 3 d after BSCTV infection. Scale bars are 10 µm. All results in this figure are representative of three independent experiments.

The C4 or AC4 proteins of most geminiviruses are localized to the membrane, although our previous study had shown that the N-terminal GFP/YFP fusion of the C4 protein from BSCTV was distributed in the cytosol of plant cells (Teng *et al.*, 2010). When YFP was fused to the C terminus of BSCTV C4, the fluorescence signals were predominantly observed on the plasma membrane of the plant protoplasts (Fig. 1A), implying that the N-terminal fusion interferes with the membrane localization of the protein. The bioinformatics software CSS-Palm (Ren *et al.*, 2008) predicted that the eighth residue on BSCTV C4 is a cysteine (C8) that is highly available for S-acylation. Cysteine residues are also found in the N termini of other geminiviruses (see Supplementary Fig. S1 at *JXB* online), suggesting a potential conserved mechanism among these virus species. An acyl-RAC assay (Lai *et al.*, 2015) was used to provide biochemical evidence for the S-acylation of BSCTV C4. The C4 protein was pulled-down on Thiopropyl Sepharose in the presence of NH_2_OH (Fig. 1B), suggesting that it was S-acylated in plant cells. Because 2-bromopalmitate (2-BP) is an inhibitor of S-acylation (Jennings *et al.*, 2009), it was used to determine the specificity of this assay. The modification of C4 was found to be sensitive to 2-BP, providing further support for the S-acylation of C4. Consistently, the membrane localization of C4-YFP was disrupted in the presence of 2-BP (Fig. 1C), suggesting that S-acylation was essential for the membrane association of C4. The S-acylation levels of C4 were also monitored during the BSCTV infection of plant cells. Compared with the vector control, BSCTV infection did not alter the S-acylation or subcellular localization of C4 (Fig. 1D, E).

**Fig. 2.**
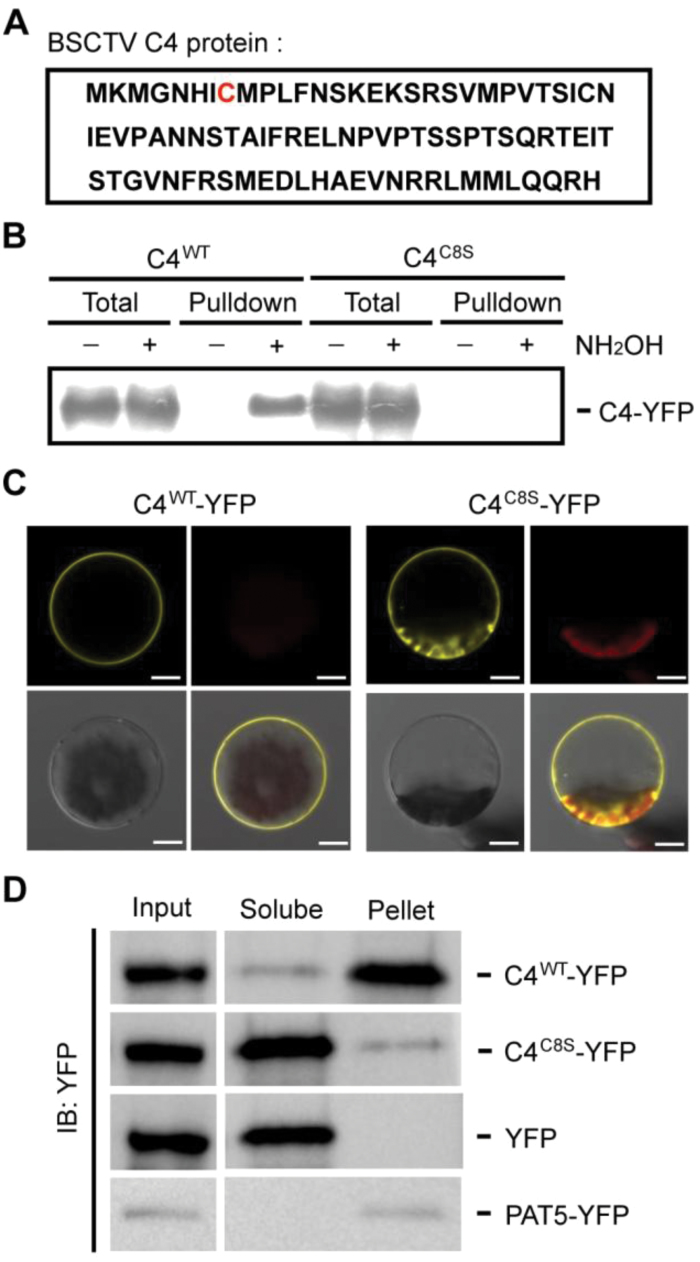
Identification of the S-acylation site in BSCTV C4. (A) The predicted S-acylation site in BSCTV C4. The amino acid sequence of C4 is shown and the potential S-acylation site (C8) is highlighted in red. (B) Characterization of the S-acylation site on the C4 protein using acyl-resin-assisted capture assays. The wild-type (WT) or C8S (cysteine to serine) mutant version of *C4-YFP-FLAG*_*3*_*His*_*6*_ was expressed in protoplasts. The total lysate is shown as the input control, and pulldown indicates the S-acylation proteins captured on Thiopropyl Sepharose. The signals were detected using immunological blotting with an anti-FLAG antibody. (C) The localization of the WT or C8S version of C4-YFP-FLAG_3_His_6_, detected using confocal microscopy. The YFP signal (yellow), chloroplast autofluorescence (red), bright field (gray), and merged images are shown. Scale bars are 10 µm. (D) Protoplasts expressing the WT or C8S version of *C4-YFP-FLAG*_*3*_*His*_*6*_ were fractionated into soluble and pellet fractions. YFP and PAT5-YFP (a transmembrane protein) were used as controls. The signals were measured from an immunological blot using an anti-GFP antibody. All results in this figure are representative of three independent experiments.

The putative residue for S-acylation, C8, was mutated from a cysteine to a serine (C8S) (Fig. 2A). An acyl-RAC assay was used to determine that this mutation resulted in the complete loss of BSCTV C4 S-acylation (Fig. 2B), indicating that this residue is the major S-acylation site on the viral protein. To confirm the requirement for S-acylation in the localization of C4, the fluorescence signals from plant cells expressing the wild-type or mutated versions of C4-YFP were detected. The results showed that mutating the C8 S-acylation site impaired the membrane association of C4 (Fig. 2C), consistent with our results from the 2-BP treatment. The S-acylation-mediated regulation of the subcellular localization of C4 was confirmed using a cell fraction assay. Similar to the transmembrane protein PAT5 (Protein S-Acyltransferase 5) (Batistič, 2012), the wild-type C4 was predominantly localized in the membrane fraction. When the S-acylation site was mutated, most C4 proteins were detected in the soluble fraction, with only a small amount of mutated protein associated with the membrane (Fig. 2D), supporting the results of the microscopy observations. The mutation of the other C4 cysteine residue, C28, did not affect the S-acylation or localization of the protein (Supplementary Fig. S2), confirming that only the eighth residue was S-acylated on C4.

Because myristoylation at the N terminus of proteins may regulate their S-acylation, the potential myristoylation site (G4) on BSCTV C4 was also mutated from a glycine to an alanine residue, which disrupted both the S-acylation and membrane association of C4 (Supplementary Fig. S3). The N terminus of BSCTV C4 contains two more residues than the C4 proteins of other geminiviruses (Supplementary Fig. S1). To exclude their effect on S-acylation, the first two residues of C4 were deleted (C4D2); however, the patterns of S-acylation and localization of C4D2 were similar to those of the full-length protein (Supplementary Fig. S4), suggesting that the S-acylation of BSCTV C4 was not dependent on the extra residues at its N terminus.

### S-acylation of C4 is critical for its function as a symptom determinant

**Fig. 3.**
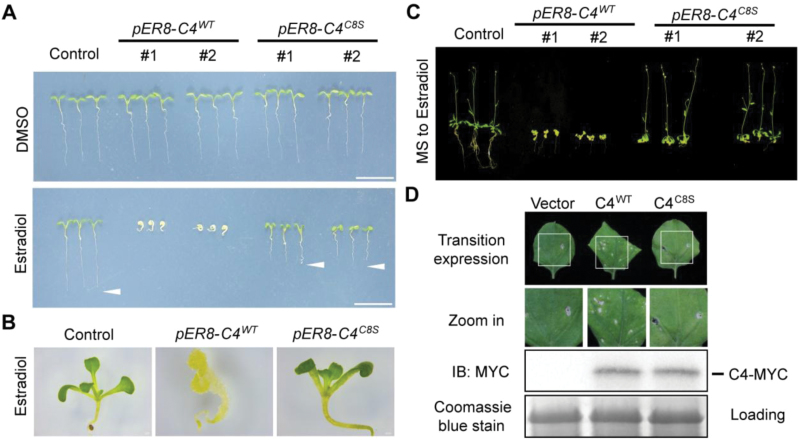
S-acylation is important for the effect of C4 on Arabidopsis development. (A) Seedlings of estradiol-inducible *pER8-C4*^*WT*^ (wild-type) and *pER8-C4*^*C8S*^ (C8 mutated from cysteine to serine) plants germinated on media with (bottom panel) or without (top panel) 2 µM estradiol for 6 d. Scale bars are 1 cm. (B) Shoot phenotypes of control, *pER8-C4*^*WT*^, and *pER8-C4*^*C8S*^ plants germinated on a medium containing 2 µM estradiol. (C) Seedlings were germinated and grown on regular medium for 7 d, then transferred onto a medium containing 2 µM estradiol for 3 weeks. The images are from independent experiments using individual transgenic lines, which produced similar patterns. (D) Agrobacteria carrying *pCanG-MYC* (Vector), *pCanG-C4*^*WT*^*-MYC*, or *pCanG-C4*^*C8S*^*-MYC* were injected into *Nicotiana benthamiana* leaves. After 4 d, their phenotypes were observed and the levels of C4 proteins were detected with using immunological blotting with an anti-MYC antibody. Coomassie Blue staining of total proteins was used as a loading control. WT, wild-type. All results in this figure are representative of at least three independent experiments.

Because C4 is a symptom determinant in BSCTV, the wild-type or C8S versions of *C4* were expressed in Arabidopsis to elucidate the effect of S-acylation on C4 function in the host plants. Transgenic plants constitutively expressing BSCTV *C4* could not be obtained because of their ectopic cell division (Lai *et al.*, 2009); therefore, the *C4* gene was cloned into the *pER8* vector and its expression was controlled using an inducible promoter (Zuo *et al.*, 2000). In the presence of estradiol, the expression of both the wild-type and C8S versions of *C4* were induced at similar levels in their respective transgenic lines (Supplementary Fig. S5). Seeds were germinated on media with or without the inducer to compare the effects of C4 on plant growth (Fig. 3A). The development of the transgenic plants without the induced *C4* expression was normal, but in the presence of estradiol, plant growth was completely inhibited by the wild-type C4, consistent with previous results (Lai *et al.*, 2009). The mutation of the S-acylation site dramatically attenuated the abnormal host development caused by C4, demonstrating the importance of S-acylation on the function of the viral protein.

In the *pER8-C4*^*C8S*^ plants, root development was reduced and the phenotypes of the hypocotyls and cotyledons were also slightly altered, while the development of the shoot was normal (Fig. 3B). To further explore the effects of C4 on shoot development, 1-week-old seedlings were transferred from regular medium to inducing medium for *C4* expression. In comparison with the *pER8-C4*^*WT*^ plants, which had reduced shoot growth, no abnormal effects on shoot development were observed in the *pER8-C4*^*C8S*^ plants (Fig. 3C), indicating that S-acylation was critical for the function of C4 in the regulation of the shoot meristem. The function of S-acylation in the symptom development mediated by C4 was also supported by a transient expression assay in *N. benthamiana* leaves. When similar levels of proteins were expressed, the wild-type C4 induced leaf curling and cell death, but this effect was impaired when the S-acylation site was mutated (Fig. 3D, Supplementary Fig. S6).

**Fig. 4.**
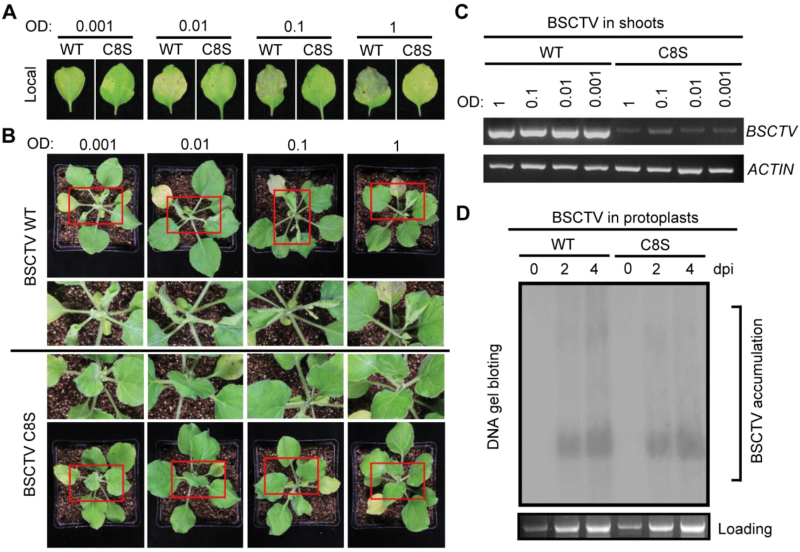
S-acylation is necessary for the function of C4 in symptom determination during BSCTV infection. (A) *Nicotiana benthamiana* plants were inoculated with *pCAMBIA1300-BSCTV-C4*^*WT*^*-1.8copy* (wild-type) or *pCAMBIA1300-BSCTV-C4*^*C8S*^*-1.8copy* (C8 mutated from a cysteine to a serine) using different concentrations of *Agrobacterium* relative to OD_600_. The leaves local to the inoculation site were photographed after 9 d. (B) The top-curling phenotype of *N. benthamiana* induced by infection with BSCTV. The plants were photographed 9 d after inoculation. Enlarged images of the shoot tips are shown for a better comparison. The top-curling symptom did not appear on the plants infected with BSCTV-C4^C8S^ even a long time after inoculation. (C) Levels of BSCTV in the newly emerged shoots of *N. benthamiana* plants were detected using PCR 9 d after inoculation. *ACTIN* (*N. benthamiana*) was used as a control. (D) Accumulation of BSCTV in Arabidopsis protoplasts was measured 0, 2, and 4 d after infection. The DNA was extracted and subjected to a DNA gel blot using the BSCTV genome as a probe. The plant genomic DNA was used as the loading control. dpi, days post-infection; WT, wild-type. The results in this figure are representative of at least three independent experiments.

Next, we investigated the role of C4 S-acylation in the alteration of host shoot development during BSCTV infection. BSCTV carrying wild-type or C8S C4 (which did not alter the amino acid sequence of the overlapping Rep protein) was used to inoculate *N. benthamiana* leaves (Teng *et al.*, 2010). Host cell death was induced by the wild-type virus, resulting in a top-curling phenotype, but not by the virus containing the mutated C4 (Fig. 4A, B). Compared with the wild-type virus, much lower levels of the C4^C8S^ virus were detected in the newly emerged shoots (Fig. 4C, Supplementary Fig. S7); however, the accumulation of both types of viruses were similar in the protoplasts and the leaves local to the inoculation site (Fig. 4D, Supplementary S7). These results suggested that S-acylation of C4 may regulate the movement but not the replication of the virus, consistent with previous results from the depletion of BSCTV C4 (Teng *et al.*, 2010). Although the accumulation of the mutated virus was detectable in the shoot region, the plants showed no symptoms even a long time after inoculation (Fig. S8), supporting the conclusion that the S-acylation of C4 was critical for symptom determination during BSCTV infection.

### S-acylation of C4 is necessary for its interaction with CLV1

**Fig. 5.**
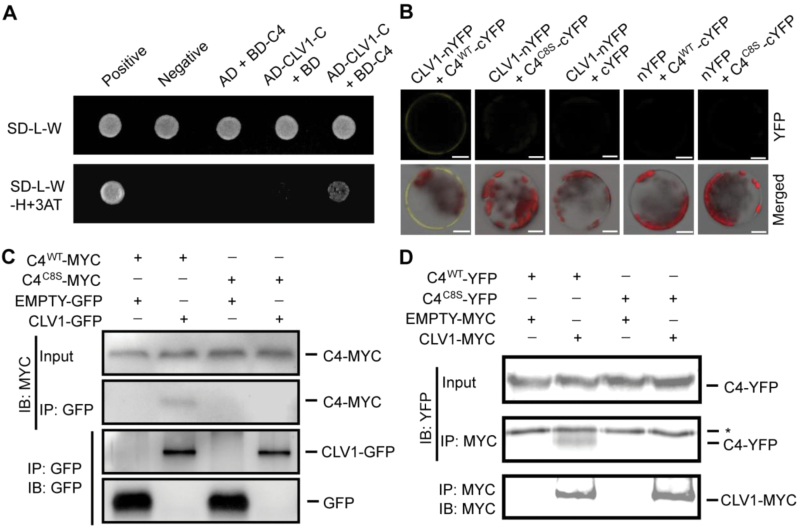
S-acylation of C4 is important for its interaction with CLV1. (A) Detection of the interaction between C4 (fused to the binding domain, BD) and the intracellular domain (704AA-967AA) of CLV1 (CLV1C; fused to the activation domain, AD) in a yeast two-hybrid assay. Protein interactions were tested using a stringent (SD/–Leu/–Trp/–His) selection medium containing 3-amino-1,2,4-triazole (3AT). (B) The *in vivo* interaction between C4 and CLV1 was measured using bimolecular fluorescence complementation assays. The combinations with empty vectors were used as negative controls. The YFP signals and merged signals (yellow for YFP, red for chloroplast autofluorescence, and gray for bright field) are shown. Scale bars are 10 µm. (C) Detection of the interaction between C4-MYC and CLV1-GFP using co-immunoprecipitation (Co-IP). The wild-type (WT) or mutant form of *pCanG-C4-MYC* (C8 mutated from a cysteine to a serine) was co-transformed with *35S::CLV1-GFP* or *35S::GFP* (control). After 48 h, the transformed protoplasts were collected for Co-IP using GFP-Trap resin. (D) Measurement of the interaction between C4-YFP-FLAG_3_His_6_ and CLV1-MYC using Co-IP. The wild-type or mutant form of *C4-YFP-FLAG*_*3*_*His*_*6*_ was co-expressed with *pCanG-CLV1-MYC* or *pCanG-MYC* (control). Co-IP was performed using an anti-MYC antibody with Protein A resins. * indicates the unspecific signal from the antibody. The results in this figure are representative of independent experiments.

Our data showed that S-acylation was important for the effect of C4 on shoot development; therefore, we next attempted to uncover the potential mechanism regulating this process. We hypothesized that the target of C4 may be associated with the plasma membrane and may play a role in regulating shoot development. A previous study showed that the intracellular catalytic domain of a leucine-rich repeat receptor-like kinase (At3g24240) interacts with BCTV C4 in a Y2H assay (Piroux *et al.*, 2007). Using a bioinformatics analysis, we found that the amino-acid sequence of the interaction domain of this protein was highly similar to that of CLAVATA 1 (CLV1), a receptor kinase involved in the regulation of the shoot meristem (Clark *et al.*, 1997; Hazak and Hardtke, 2016). A Y2H assay revealed that BSCTV C4 interacted with the intracellular catalytic domain of CLV1 in yeast cells (Fig. 5A).

Next, we assessed this interaction in plant cells and investigated whether it was dependent on the S-acylation of C4. First, the results from a BiFC assay showed that full-length CLV1 and C4 interacted on the plasma membrane *in vivo*, but that the depletion of S-acylation disrupted this association (Fig. 5B). This interaction was further confirmed by performing Co-IP assays in plant cells, which showed that CLV1 specifically interacted with wild-type C4 but not with the mutated C4 (Fig. 5C, D), providing direct evidence that their interaction was mediated by S-acylation in plant cells.

### C4-induced misexpression of *WUS* is dependent on S-acylation

**Fig. 6.**
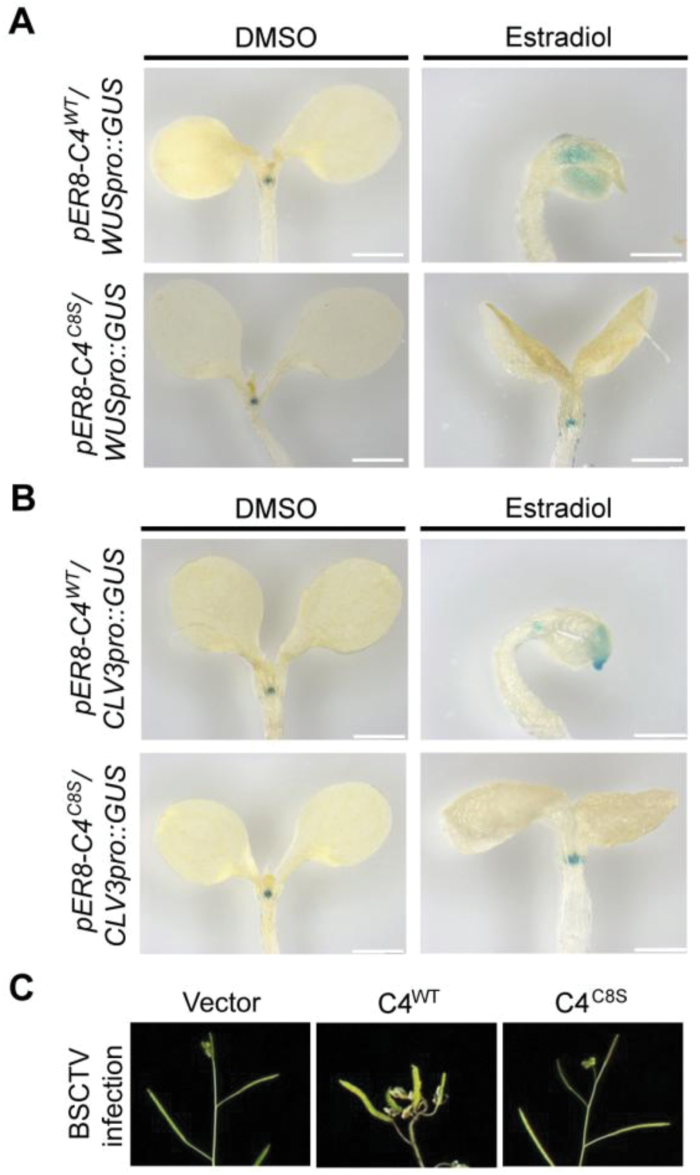
C4-induced misexpression of *WUS* is dependent on its S-acylation. (A) GUS staining levels of *WUSpro::GUS* in the estradiol-inducible *pER8-C4*^*WT*^ (wild-type) and *pER8-C4*^*C8S*^ (C8 mutated from a cysteine to a serine) plants 5 d after germination on media with (estradiol) or without (DMSO) induction by estradiol. Scale bars are 0.5 mm. (B) GUS staining levels of *CLV3Pro::GUS* in the *pER8-C4*^*WT*^ and *pER8-C4*^*C8S*^ plants 5 d after germination on media with or without estradiol. Scale bars are 0.5 mm. The results are representative of three independent experiments using individual transgenic lines, which had similar patterns. (C) Representative siliques of Arabidopsis infected with the geminivirus carrying the wild-type (WT) or C8S version of C4. The empty vector was used as an inoculation control.

CLV1 is a receptor-like kinase that perceives the secreted peptide CLV3 on the plasma membrane, then negatively regulates the expression of the homeodomain transcription factor *WUSCHEL* (*WUS*) to modulate cell differentiation in the shoot and floral meristems (Brand *et al.*, 2000). The interaction between C4 and CLV1 may therefore interfere with this signaling transduction. To detect the expression of *WUS*, a *GUS* gene under the control of the *WUS* promoter was crossed into *pER8-C4*^*WT*^ and *pER8-C4*^*C8S*^ plants. *WUS* was expressed in the shoot-meristem region of the seedlings in the absence of *C4* expression (Fig. 6A). The pattern of GUS staining was dramatically changed in the presence of *C4* expression and showed that the expression of *WUS* was inhibited in the meristem but enhanced in the cotyledons, suggesting the abnormal regulation of this pathway by the viral protein. By contrast, the expression of *WUS* was not affected by the C4^C8S^ mutant protein, supporting the role of S-acylation in this process.

The WUS protein migrates into adjacent cells to activate *CLV3* expression and establish a feedback loop to balance the number of stem cells produced with the size of the meristem (Yadav *et al.*, 2011); therefore, we also measured the transcription levels of *CLV3* in the presence of C4. The expression of *CLV3* was detectable but weaker in the meristems in the presence of wild-type C4, but it also abnormally occurred in the cotyledons (Fig. 6B). This result was consistent with feedback regulation and provided evidence that the inhibition of *WUS* expression by C4 was not a result of a misorganization of the shoot meristem; therefore, C4 may interact with CLV1 on the plasma membrane following its S-acylation, causing it to interfere with the CLAVATA pathway.

The mutation of components involved in the Arabidopsis CLAVATA pathway often causes phenotypic changes in the siliques (Somssich *et al.*, 2016), so those on the BSCTV-infected plants may also have abnormal cell division. We found that infection with BSCTV carrying the wild-type but not the C8S mutant form of C4 induced abnormal development in the siliques in Arabidopsis (Fig. 6C), providing further evidence of a potential association between BSCTV infection and the CLAVATA pathway.

## Discussion

Our current study revealed a novel mechanism for the role of S-acylation in the interaction between geminiviruses and plants. Certain viral proteins are regulated by S-acylation in mammalian host cells; for instance, S-acylation is essential for the association of the influenza virus haemagglutinin protein with the membrane microdomains of the animal cell (Veit *et al.*, 2013). Similarly, the S-acylation of C4 regulates its localization to the plasma membrane of the plant cell and thus its consequential cellular effects. Our results showed that the S-acylation level of C4 was not altered during BSCTV infection (Fig. 1). When *C4* is expressed in infected plant cells, the protein may be recognized and S-acylated by the host cell; however, this modification contributes to the efficiency of the viral infection, implying a functional interaction between pathogen and host during evolution.

It has been reported that myristoylation is also involved in the localization of geminivirus C4 proteins to the host membrane (Fondong *et al.*, 2007; Piroux *et al.*, 2007). Unlike myristoylation, S-acylation is reversible and causes the modified protein to have a higher affinity with the membrane, potentially providing a dynamic regulation of its membrane association (Linder and Deschenes, 2007). The mutation of the myristoylation site of C4 in BCTV resulted in the complete inactivation of this pathogenesis determinant (Piroux *et al.*, 2007); here, we showed that S-acylation on BSCTV C4 was important for its function in regulation of shoot symptoms. Interestingly, in the current study, the mutation of the potential myristoylation site at the N terminus of BSCTV C4 disrupted its S-acylation and membrane localization (Supplementary Fig. S3), supporting the hypothesis that myristoylation may have consequential effects on S-acylation. The results from the cellular fraction showed that most C4^C8S^ proteins were distributed in the cytosol, but a small amount of mutated proteins were retained at the membrane (Fig. 2), possibly mediated by myristoylation or physical interaction with other membrane proteins. This small proportion of C4 proteins may be sufficient to influence root development, which may be more sensitive to C4 (Fig. 3). Because S-acylation is reversibly regulated by S-acyltransferases and thioesterases (Lanyon-Hogg *et al.*, 2017), the modification level of C4 may be dynamically controlled depending on the cellular conditions. There are 24 S-acyltransferases in Arabidopsis (Batistič, 2012) and many potential thioesterases, so it would be interesting to identify the specific enzymes involved in the regulation of C4 S-acylation. This would enhance our understanding of the precise mechanism controlling this symptom determinant.

Our results showed that the S-acylation of C4 did not have an effect on virus replication (Fig. 4), but may have contributed to virus movement, consistent with the previously described function of this protein (Teng *et al.*, 2010). Although the movement of viruses to the newly emerged shoots was slower when the S-acylation site of C4 was mutated, BSCTV was still detectable in this region; however, even a long time after inoculation, the mutant BSCTV did not cause obvious symptoms in the shoots (Supplementary Fig. S8), supporting our conclusion that S-acylation is important for symptom development during BSCTV infection. The C4 proteins of several geminiviruses were previously identified as suppressors of gene silencing (Ramesh *et al.*, 2017), so future research should investigate whether S-acylation is involved in the regulation of this process.

The identification of the interaction between C4 and CLV1 provided a potential mechanism to explain the effects of C4 on shoot development. Although the data from Y2H assays indicated that C4 interacted with the intracellular domain of CLV1, the Co-IP results using full-length proteins suggested that membrane localization of C4 mediated by S-acylation was critical for this interaction in plant cells (Fig. 5). Because CLV1 is a transmembrane protein, S-acylation of C4 may enhance its interaction with CLV1 in the membrane regions, affecting the perception of the CLV3 peptide for the downstream repression of *WUS* expression (Hazak and Hardtke, 2016). Our data showed that C4 inhibited *WUS* expression in the meristem regions but stimulated it in the cotyledons (Fig. 6), suggesting that this protein interferes with the CLAVATA signaling pathway. Membrane biochemistry is technologically difficult to study, but future investigations of the precise mechanisms by which C4 regulates CLV1 may yield interesting results. Geminivirus infections result in the abolishment of meristems and induce ectopic cell division in differentiated tissues (Park *et al.*, 2002), which is consistent with our data showing abnormal *WUS* expression in the presence of C4. BSCTV induced cell division in the shoot tips of the plants, including the siliques, which is a similar phenotype to those of plants with mutations in components of the CLAVATA pathway; therefore, the CLAVATA signaling pathway may contribute to these symptoms. Previous studies indicated that BCTV C4 interacts with AtSKs and BIN2 (Mills-Lujan *et al.*, 2015; Bi *et al.*, 2017), interfering with BR signaling during infection. It will be also important to determine whether there is a C4-related connection between CLAVATA and BR signaling. In addition, C4 proteins from some geminiviruses are phosphorylated (Mills-Lujan *et al.*, 2015). S-acylation is critical for the interaction between BSCTV C4 and CLV1, which is a receptor kinase; thus, it would be interesting to examine the relationship between the effects of S-acylation and phosphorylation on BSCTV C4.

Although the protein sequences of geminivirus C4 proteins vary, cysteine residues are found in many members (Supplementary Fig. S1), suggesting a potential conserved mechanism mediated by S-acylation. The S-acylation of this pathogen determinant has not yet been biochemically characterized, however. Different geminiviruses cause differing symptoms, and their regulatory mechanisms may vary. In a previous study on myristoylation of AC4 from the East African cassava mosaic Cameroon virus (EACMCV), it was shown that a cysteine mutation did not apparently affect its function in pathogenesis (Fondong *et al.*, 2007), implying that not all cysteines are essential for virus infection. In future studies, it would be interesting to investigate C4 conservation and variation following S-acylation in different types of geminiviruses, which could improve our understanding on the role of protein modifications in the interaction between viruses and plant cells.

## Supplementary data

Supplementary data are available at *JXB* online.

Table S1. Primers used in this study.

Fig. S1. Sequence alignment of C4 proteins from different geminiviruses.

Fig. S2. Localization and S-acylation analysis of the C4^C28S^ mutant.

Fig. S3. Localization and S-acylation analysis of the C4^G4A^ mutant.

Fig. S4. Localization and S-acylation analysis of the C4D2 mutants.

Fig. S5. The expression levels of *C4* in transgenic plants.

Fig. S6. Functional analysis of the C4^C8A^ mutant in *N. benthamiana* leaves.

Fig. S7. The effect of C4 S-acylation on BSCTV accumulation in local inoculated leaves and newly emerged shoots.

Fig. S8. The effect of C4 S-acylation on symptoms at 2 weeks and 3 weeks after BSCTV inoculation.

Supplementary Tables and Figures
