# Supplementary material for: S-acylation of a geminivirus C4 protein is essential for regulating the CLAVATA pathway in symptom determination
Source: J Exp Bot. 2018 Jun 20;69(18):4459–68. doi: 10.1093/jxb/ery228 (PMC6093331; doi:10.1093/jxb/ery228)
Supplement: Supplementary Tables and Figures [file ery228_suppl_supplementary_tables_figures.pdf]

S-acylation of a geminivirus C4 protein is essential for regulating the CLAVATA pathway in symptom determination

Huiyun Li[1,†], Runxiu Zeng[1,†], Zian Chen[1,†], Xiaoshi Liu[1], Zhendan Cao[1], Qi Xie[2], Chengwei Yang[1] and Jianbin Lai[1,*]

[1] Guangdong Provincial Key Laboratory of Biotechnology for Plant Development, School of Life Science, South China Normal University, Guangzhou 510631, China

[2] Institute of Genetics and Developmental Biology, Chinese Academy of Sciences, Beijing 100101, China

[†] These authors contributed equally to this work.

*Correspondence: 20141062@m.scnu.edu.cn

Supplementary data

Table S1. Primers used in this study.

Fig. S1. Sequence alignment of C4 proteins from different geminiviruses.

Fig. S2. Localization and S-acylation analysis of the $C4^{C28S}$ mutant.

Fig. S3. Localization and S-acylation analysis of the $C4^{G4A}$ mutant.

Fig. S4. Localization and S-acylation analysis of the C4D2 mutants.

Fig. S5. The expression levels of *C4* in transgenic plants.

Fig. S6. Functional analysis of the $C4^{C8A}$ mutant in *N. benthamiana* leaves.

Fig. S7. The effect of C4 S-acylation on BSCTV accumulation in local inoculated leaves and newly emerged shoots.

Fig. S8. The effect of C4 S-acylation on symptoms at 2 weeks and 3 weeks after BSCTV inoculation.

Table S1. The primers used in this study.

C4-YFP-F	AGTGGATCCATGAAAATGGGGAACCACATCTG
C4-YFP-R	AGTCCATGGCATGCCTCTGCTGCAGCATCATTAG
C4C8S-YFP-F	AGTGGATCCATGAAAATGGGGAACCACATCTCCATGCCCTTATTC
PER8-C4-C8S-F	GTCTCGAGCCCGGGAATTCGATTCATATGAAAATGGGGAACCACATC
PER8-C4-C8S-R	ACTACTAGTGATTGTCGACTTAATGCCTCTGCTGCAGCATC
C4-MYC-F	AGTTCTAGAATGAAAATGGGGAACCACATC
C4-MYC-R	AGTCTCGAGAATGCCTCTGCTGCAGCATC
BSCTV-C4-C8S-F	AATGGGGAACCACATCTCCATGCCCTTATTCAATTC
BSCTV-C4-C8S-R	TTCATGTAATTCTCTGCAGATG
BD-C4-F	AGTGAATTCATGAAAATGGGGAACCACATCTG
BD-C4-R	AGTGGATCCTTAATGCCTCTGCTGCAGCATC
AD-CLV1C-F	AGTGAATTCATTGTCTACCGTGGATCAATGCC
AD-CLV1C-R	AGTGGATCCTCAGAACGCGATCAAGTTCGCC
CLV1-MYC-F	GAGAACACGGGGGACTCTAGAATGGCGATGAGACTTTTGAAG
CLV1-MYC-R	TCTGTACAGGCGCGCCTCGAGAGAACGCGATCAAGTTCGCCAC
CLV1-GFP-F	AGTCCATGGCGATGAGACTTTTGAAGACTCATCTTC
CLV1-GFP-R	AGTGGTACCGAACGCGATCAAGTTCGCCACGGAT
C4-G4A-F	AATTACAGTCGAGGGGGATCCATGAAAATGGCGAACCACATC
C4-C28S-F	TGCCCGTTACTTCGATCAGCAACATCGAAGTACCAG
C4-C28S-R	TTACGGATCTGGACTTTTCCTTC
C4D2-F	AATTACAGTCGAGGGGGATCCATGGGGAACCACATCTGCATG
C4-C8A-F	AGTTCTAGAATGAAAATGGGGAACCACATCGCCATGCCCTTATTCAATTCG
C4-RT-F	TCCAGATCCGTAATGCCCGT
C4-RT-R	GAGACGTAGGACTTGACGTCG
ACTIN-RT-F	TGGAATGGTGAAGGCTGGAT
ACTIN-RT-R	CCAGTTGTACGACCACTAGC

Fig.S1

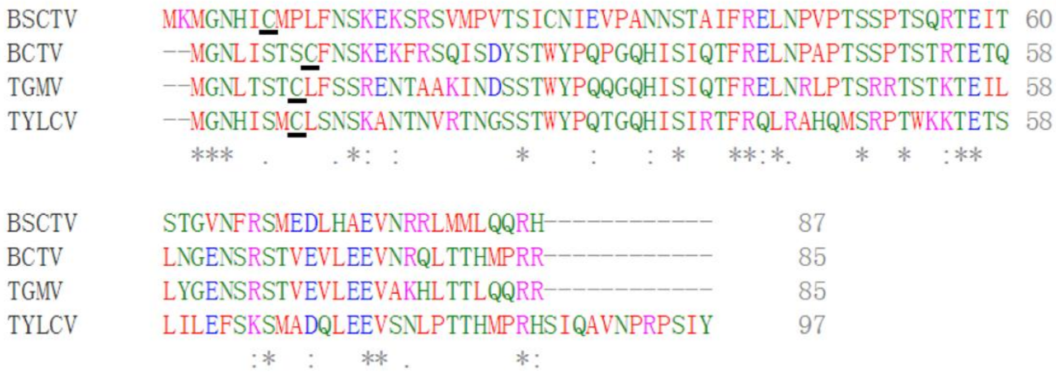

Fig. S1. Sequence alignment of C4 proteins from different geminiviruses.

The protein sequences of C4 proteins from different geminiviruses, including BSCTV, BCTV, Tomato golden mosaic virus (TGMV) and Tomato yellow leaf curl virus (TYLCV), were aligned using the Clustal software. The conserved residues are shown in Clustal color code. The cysteine residues at the N terminus of these proteins are labeled by black lines.

Fig.S2

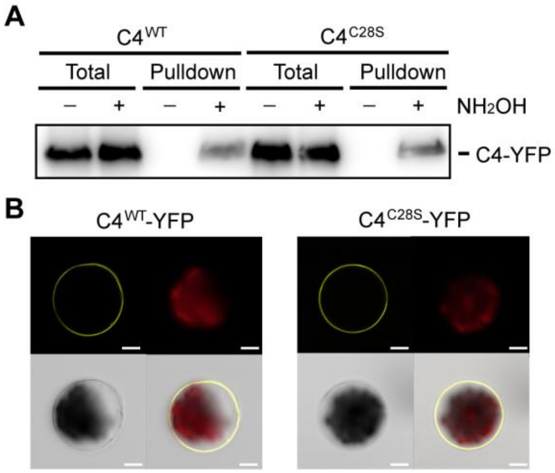

Fig. S2. Localization and S-acylation analysis of the C4^{C28S} mutant.

(A) Measurement of S-acylation on the wild-type and C28S mutant of C4 proteins. (B) The localization of C4^{WT}-YFP-FLAG$_3$His$_6$ and C4^{C28S}-YFP-FLAG$_3$His$_6$. The YFP signal (yellow), chloroplast autofluorescence (red), bright field (gray), and merged images are shown. Bar = 10 μm. The data are representative of independent experiments.

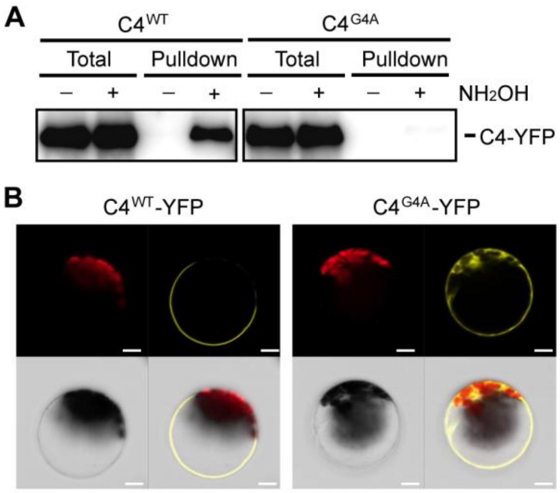

Fig. S3. Localization and S-acylation analysis of the C4^{G4A} mutant.

(A) Measurement of S-acyaltion on the wild-type and G4A mutant of C4 proteins. (B) The localization of C4^{WT}-YFP-FLAG$_3$His$_6$ and C4^{G4A}-YFP-FLAG$_3$His$_6$. The YFP signal (yellow), chloroplast autofluorescence (red), bright field (gray), and merged images are shown. Bar = 10 μm. The data are representative of independent experiments.

Fig.S4

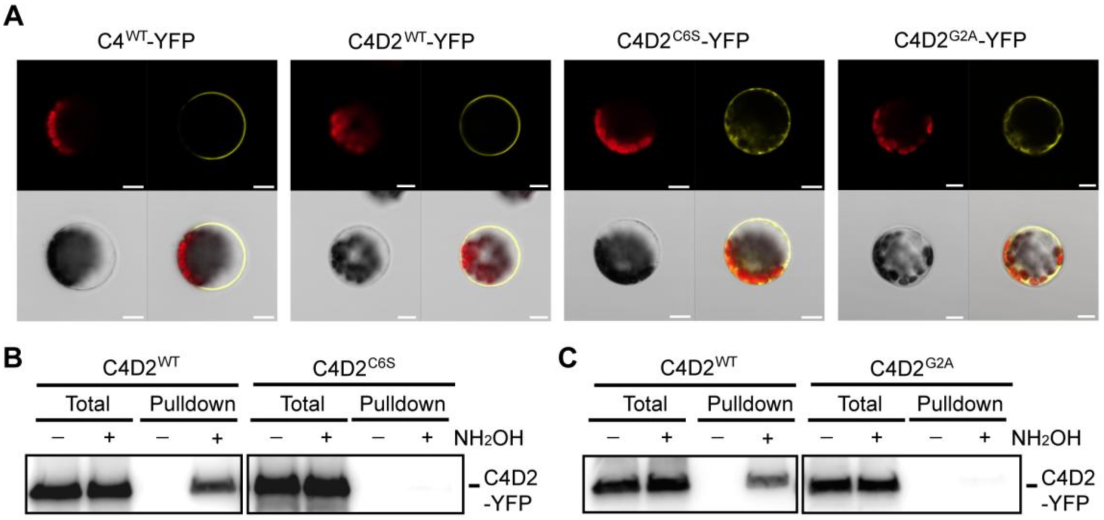

Fig. S4. Localization and S-acylation analysis of the C4D2 mutants.

(A) The localization of C4^{WT}-YFP-FLAG$_3$His$_6$, C4D2^{WT}-YFP-FLAG$_3$His$_6$, C4D2^{C6S}-YFP-FLAG$_3$His$_6$ or C4D2^{G2A}-YFP-FLAG$_3$His$_6$ was detected under confocal microscope. C4D2 indicates the version of BSCTV C4 without first two residues. The YFP signal (yellow), chloroplast autofluorescence (red), bright field (gray), and merged images are shown. Bar = 10 μm. The data are representative of independent experiments. (B) Measurement of S-acylation on the wild-type and C6S mutant of C4D2 proteins. (C) Measurement of S-acylation on the wild-type and G2A mutant of C4D2 proteins. All the results are representative of independent experiments.

Fig.S5

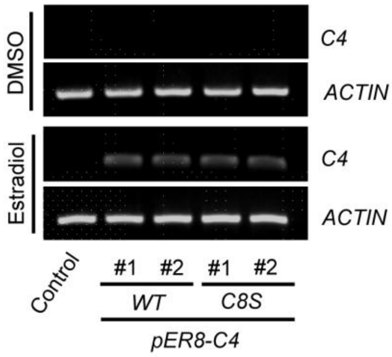

Fig. S5. The expression levels of *C4* in the transgenic plants.

The 6-day-old *pER8-C4 WT* and *C8S* transgenic plants were treated with DMSO or 2 μM estradiol for 24 hours and RNA was extracted for RT-PCR. *ACTIN* was used as a reference gene. The result is representative of three independent experiments.

Fig.S6

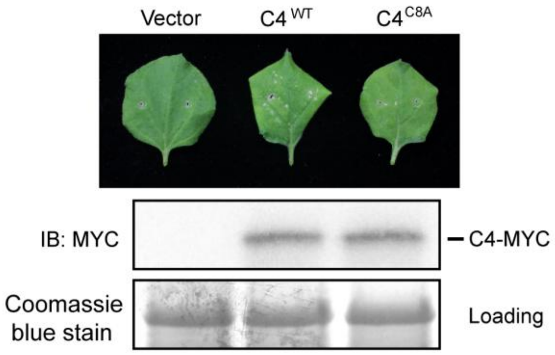

Fig. S6. Functional analysis of the C4^{C8A} mutant in *N. benthamiana* leaves.

The agrobacteria carrying *pCanG-MYC* (Vector), *pCanG-C4^{WT}-MYC* or *pCanG-C4^{C8A}-MYC* were injected into tobacco leaves for transient expression. Four days after injection, the phenotypes were observed and the expression levels of C4 proteins were detected with an immunological blot using the anti-MYC antibody. Coomassie blue staining of total proteins were used as a loading control. The result is representative of three independent experiments.

Fig.S7

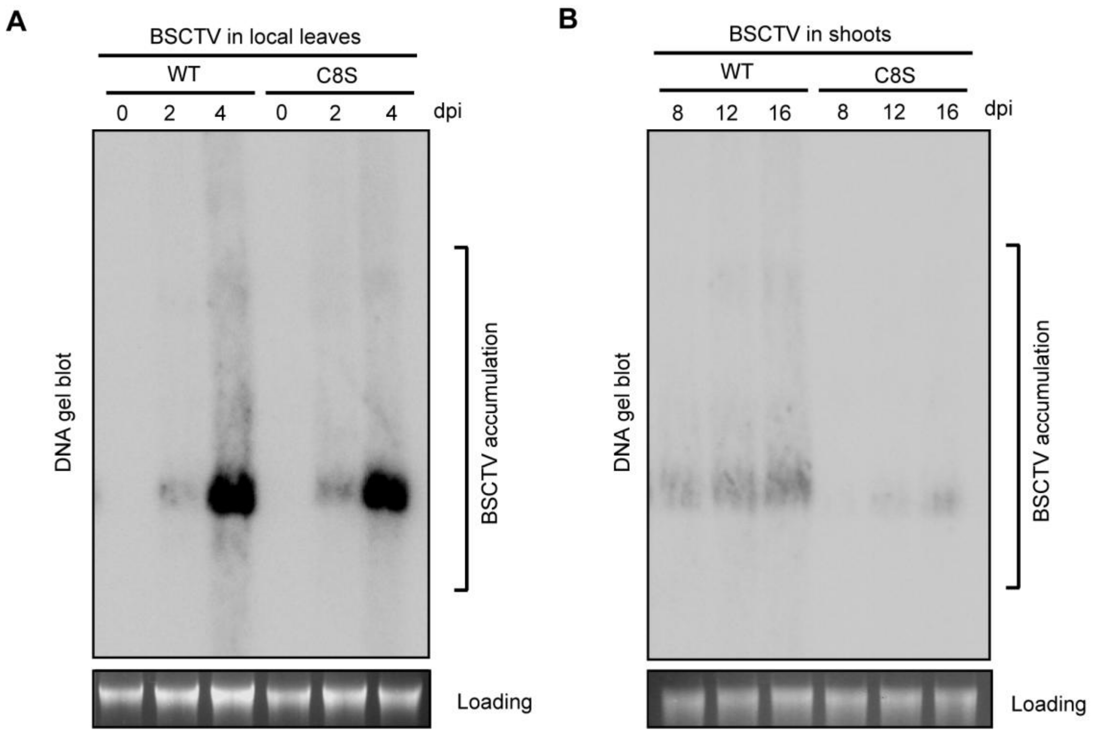

Fig. S7. The effect of C4 S-acylation on BSCTV accumulation in local inoculated leaves and newly emerged shoots.

(A) Accumulation of BSCTV in the local inoculated leaves was measured 0 day, 2 days and 4 days after infection. (B) Accumulation of BSCTV in the newly emerged shoots was detected 8 days, 12 days and 16 days after infection. DNA was extracted and subjected for DNA gel blot using the BSCTV genome as a probe. The plant genomic DNA was used as a loading control. DPI, days post infection. All the results above are representative of three independent experiments.

Fig.S8

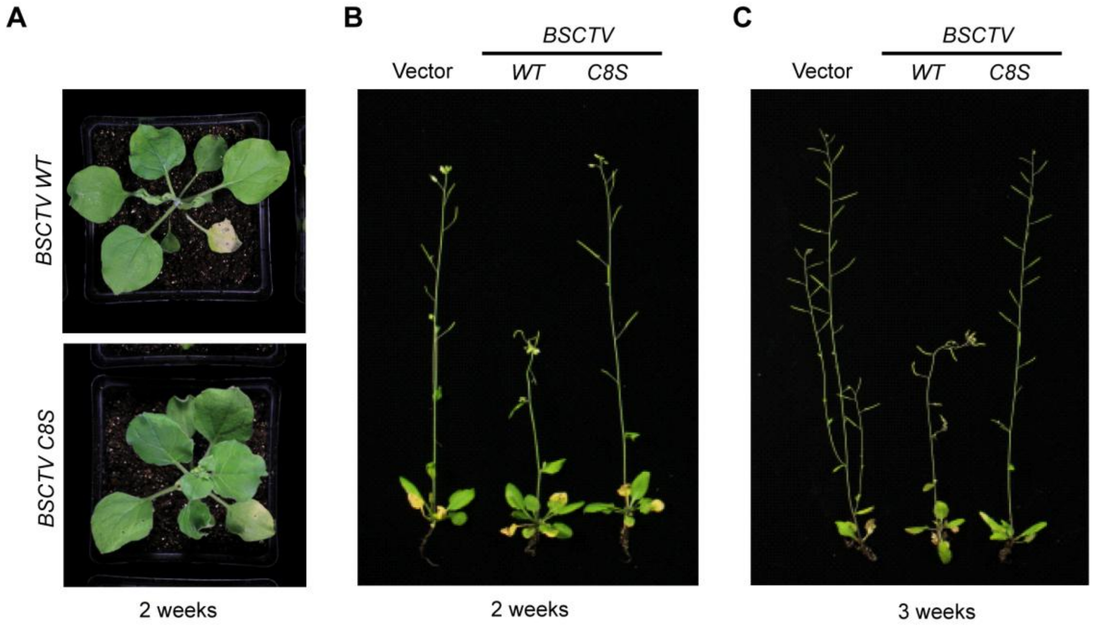

Fig. S8. The effect of C4 S-acylation on symptoms at 2 weeks and 3 weeks after BSCTV inoculation.

(A) The symptoms tobacco plants 2 weeks after inoculation using WT or $C4^{C8S}$ BSCTV (OD_{600}=0.01). (B) The symptoms of Arabidopsis 2 weeks after infection by BSCTV carrying WT or C8S version of C4. (C) The symptoms of Arabidopsis 3 weeks after infection by BSCTV carrying WT or C8S version of C4. Empty vector was used as an inoculation control. The representative plants from independent experiments are shown.
